# Multifaceted Mechanisms of Cisplatin Resistance in Long-Term Treated Urothelial Carcinoma Cell Lines

**DOI:** 10.3390/ijms19020590

**Published:** 2018-02-16

**Authors:** Margaretha A. Skowron, Margarita Melnikova, Joep G. H. van Roermund, Andrea Romano, Peter Albers, Jürgen Thomale, Wolfgang A. Schulz, Günter Niegisch, Michèle J. Hoffmann

**Affiliations:** 1 Department of Urology, Medical Faculty, Heinrich Heine University, 40225 Duesseldorf, Germany; Margaretha.Skowron@hhu.de (M.A.S.); peter.albers@med.uni-duesseldorf.de (P.A.); Wolfgang.Schulz@hhu.de (W.A.S.); Guenter.Niegisch@hhu.de (G.N.); 2 Institute of Cell Biology (Cancer Research), University of Duisburg-Essen Medical School, 45122 Essen, Germany; margarita.melnikova@uni-due.de (M.M.); juergen.thomale@uni-due.de (J.T.); 3 Department of Urology, Maastricht University Medical Centre, 6202AZ Maastricht, The Netherlands; joep.van.roermund@mumc.nl; 4 Department of Obstetrics and Gynaecology, GROW-School for Oncology & Developmental Biology, Maastricht University Medical Centre, 6229HX Maastricht, The Netherlands; a.romano@maastrichtuniversity.nl

**Keywords:** urothelial carcinoma, cisplatin, resistance mechanisms, metallothionein, Survivin, YM155

## Abstract

Therapeutic efficacy of cisplatin-based treatment of late stage urothelial carcinoma (UC) is limited by chemoresistance. To elucidate underlying mechanisms and to develop new approaches for overcoming resistance, we generated long-term cisplatin treated (LTT) UC cell lines, characterised their cisplatin response, and determined the expression of molecules involved in cisplatin transport and detoxification, DNA repair, and apoptosis. Inhibitors of metallothioneins and Survivin were applied to investigate their ability to sensitise towards cisplatin. Cell growth, proliferation, and clonogenicity were examined after cisplatin treatment by MTT 3-(4,5-dimethylthiazol-2-yl)-2,5-diphenyltetrazolium bromide, EdU (5-ethynyl-2’-deoxyuridine) incorporation assay, and Giemsa staining, respectively. Cell cycle distribution and apoptosis were quantified by flow cytometry. mRNA and protein expressions were measured by real-time quantitative (qRT)-PCR, western blot, or immunofluorescence staining. LTTs recovered rapidly from cisplatin stress compared to parental cells. In LTTs, to various extents, cisplatin exporters and metallothioneins were induced, cisplatin adduct levels and DNA damage were decreased, whereas expression of DNA repair factors and specific anti-apoptotic factors was elevated. Pharmacological inhibition of Survivin, but not of metallothioneins, sensitised LTTs to cisplatin, in an additive manner. LTTs minimise cisplatin-induced DNA damage and evade apoptosis by increased expression of anti-apoptotic factors. The observed diversity among the four LTTs highlights the complexity of cisplatin resistance mechanisms even within one tumour entity, explaining heterogeneity in patient responses to chemotherapy.

## 1. Introduction

Bladder cancer (BC) is the 9th most common tumour world-wide and the most common cancer of the urinary tract [1]. About 430,000 new bladder cancer cases and 165,000 bladder cancer deaths occurred worldwide in 2012, mostly in males [1,2]. In developed countries, about 90% of BCs are urothelial carcinomas (UC). Although cisplatin-based chemotherapy is initially efficient in UC, most patients will experience cisplatin-resistant relapses [1].

Cisplatin, following activation by aquation, initiates cell death primarily through binding to DNA at the N7 of purine bases, leading to the formation of cross-links inhibiting DNA replication and transcription, triggering cell cycle arrest and apoptosis [3,4]. The most prominent lesions are intrastrand crosslinks, which, unless repaired by nucleotide excision repair [5], lead to apoptosis [4,6].

Diverse molecular mechanisms underlying cisplatin resistance including decreased cellular uptake, increased cellular efflux, enhanced cellular inactivation, and increased cellular tolerance to DNA damage have been described for different cancer types. Thus, factors contributing to cisplatin resistance are usually categorised as mediating pre-target (transport, metabolism), on-target (DNA-cisplatin adduct formation, DNA-damage response), or post-target (evasion of apoptosis, cell-cycle arrest) resistance [6,7,8,9].

Relevant pre-target factors from the literature comprise CTR1, MT1A/B, GSH, as well as MRP and ATP7 exporters. Cisplatin is mainly internalised by the copper transporter CTR1. Accordingly, CTR1 down-regulation has been associated with resistance to platinum-based drugs in cancer [10,11]. Cellular inactivation of cisplatin and subsequent sequestration can be mediated by binding to cytoplasmic nucleophilic factors, including glutathione (GSH) and metallothioneins (e.g., MT1A and B). Overexpression of metallothioneins has been implicated in cisplatin resistance in several cancer types [12,13], including bladder cancer [14,15]. In addition, we observed increased GSH levels in most long-term cisplatin treated (LTTs) UC cell lines (UCCs) in our previous study [16]. Cisplatin–GSH conjugates are then exported by MRP2 (also known as cMOAT and ABCC2) [17]. Concurringly, overexpression of MRP transporters and concomitantly increased GSH in cisplatin-resistant cells have previously been reported [18,19]. Efflux of unconjugated cisplatin is likely mediated by the Transporting P-type Adenosine Triphosphatases ATP7A and ATP7B [20]. Overexpression of these transmembrane carriers has been implicated in cisplatin resistance and poor patient survival in oral squamous cell carcinoma [21]. ATP7B mRNA expression and IC_50_ values for cisplatin significantly correlated in 60 National Cancer Institute (NCI) cell lines [22].

As a consequence of altered cisplatin uptake and metabolism, the number of platinum adducts can be minimised. Further, on-target mechanisms like alterations in DNA damage responses have been observed in cancers. Cisplatin-induced DNA damage is mostly repaired by nucleotide excision (NER) and homologous recombination (HR) repair, or is overcome by translesion synthesis (TLS) [6]. Specifically, interesting candidates relevant for on-target resistance are ERCC1/2 (NER), MLH1, MSH2 (DNA mismatch repair, MMR), and POLH (TLS) [23,24,25,26].

Enabling a major post-target resistance mechanism, several key apoptosis-regulating factors were observed to contribute to evasion of cisplatin-induced apoptosis in cancers. Death receptors, cytoplasmic adaptors, pro- and anti-apoptotic members of the BCL2 protein family, such as BAX, BAK, BCL2, BCL-xL, and Survivin/BIRC5, caspases, calpains, and mitochondrial intermembrane proteins have been shown to modulate the post-target response to cisplatin in various cells [8,27,28]. In particular, high endogenous expression of the anti-apoptotic BCL2 family members BCL2 and BCL-xL was associated with increased cisplatin resistance in a large panel of head and neck squamous cell carcinoma cell lines [29]. Survivin has previously been identified as a crucial anti-apoptotic factor in bladder cancer, presumably by inhibiting caspases 3, 7, and 9 [30]. Its expression increases with UC progression [31] and is associated with recurrence- and progression-free survival [32].

In summary, cisplatin resistance may originate from a plethora of diverse mechanisms in different cancer types and cell lines. Thus, mechanisms identified in one cancer type may not simply be inferred as relevant in other cancers or even in other cases of the same cancer type. For a comprehensive analysis of the aforementioned factors across a panel of different UC cell lines, we established cisplatin-resistant sublines from commonly used, genetically and phenotypically different UC cell lines (UCCs) by long-term treatment (LTT) with escalating drug doses. The LTT variants displayed phenotypical changes associated with altered expression of epithelial-to-mesenchymal transition (EMT) markers and WNT-target genes, but were not enriched for subpopulations with stem-like properties that might harbour intrinsic chemoresistance [33]. Thus, our data support the idea that cisplatin resistance in UC rather originates from acquired resistance. We moreover identified NRF2 as one mediator of cisplatin resistance in UCCs, activated to different extents and by different mechanisms between the cell lines [16].

Further findings of the present study demonstrate the presence of pre-, on- and post-target mechanisms underlying cisplatin resistance, but to various degrees, in each of the four resistant UC cell lines, with a particularly important role for Survivin-dependent anti-apoptotic signalling.

## 2. Results

### 2.1. Long-Term Cisplatin Treated (LTTs) Urothelial Carcinoma Cell Lines Recover from Cisplatin-Induced Stress

**Figure 1:**
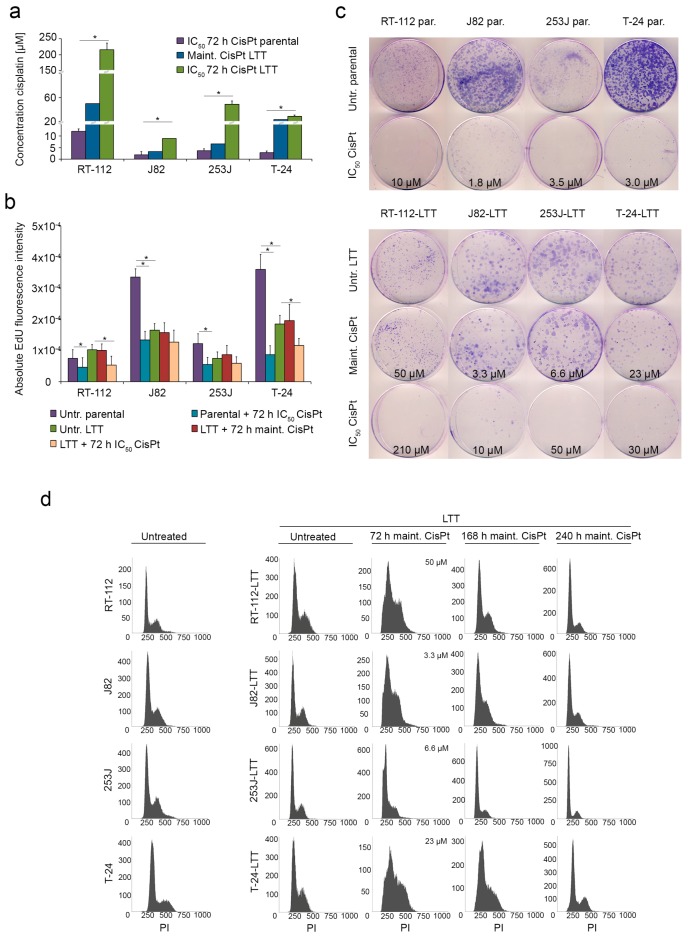
Long-term cisplatin treated cells (LTTs) recover from cisplatin-induced stress. (**a**) Cell viability was measured in treatment-naive parental urothelial carcinoma cell lines (UCCs) and resistant LTTs 72 h after treatment with indicated doses of cisplatin by MTT assay. Maintenance cisplatin concentration of LTTs is shown for comparison; (**b**) Proliferation of parental cells and LTTs treated with either IC_50_ or maintenance concentrations of cisplatin for 72 h as measured by EdU incorporation assay; (**c**) Colony formation assay of parental cells and LTTs treated with either IC_50_ or maintenance concentrations of cisplatin for 72 h. Cell clones were stained by Giemsa; (**d**) Changes in cell-cycle distribution and amount of apoptotic cells (as sub-G1 fraction) in untreated LTTs and 72, 168, and 240 h after maintenance cisplatin treatment measured by flow cytometry. Untreated parental UCCs served as a control (left panel). Values represent the mean ± standard deviation (SD) of two independent experiments. * *p* < 0.05.

Four cisplatin-resistant LTT sublines were generated from the UCCs RT-112, J82, 253J, and T-24 [16,33]. Resistance was quantified by determination of IC_50_ values following 72 h cisplatin treatment, which were between 10-fold and 21-fold increased compared to their parental controls (Figure 1a). RT-112-LTT, J82-LTT, 253J-LTT, and T-24-LTT were routinely cultured at maintenance concentrations below their IC_50_ values, but still between two-fold and eight-fold higher than the parental cell IC_50_ concentrations (Figure 1a, Table S3). Decreased EdU (5-ethynyl-2’-deoxyuridine) incorporation following treatment with IC_50_ cisplatin doses demonstrated their direct inhibitory effect on cell proliferation (Figure 1b).

Clonogenicity of parental cell lines was significantly inhibited by IC_50_ cisplatin concentrations (Figure 1c, upper part). Similar results were obtained when LTTs cells were treated with their respective, much higher IC_50_ doses. In contrast, treatment with maintenance doses did not significantly inhibit long-term proliferation capacity of LTT cells underlining their acquired cisplatin resistance (Figure 1c, lower part). Following this treatment, LTT sublines displayed typical changes in cell cycle distribution (Figure 1d), in particular accumulation of cells in S-phase, but managed to re-enter the cell cycle within 7 to 10 days to display cell cycle profiles resembling those of untreated parental cell lines as well as untreated LTTs (Figure 1d, left panels).

As in the clinic cisplatin is coadministered as a combination with other chemotherapeutic substances, cross-resistance of LTTs towards gemcitabine and doxorubicin was determined. Interestingly, a 16-fold cross-resistance to gemcitabine in RT-112-LTT and a 2.1-fold cross-resistance to doxorubicin in T-24-LTT were observed (Table S1).

### 2.2. Cisplatin Exporter and Detoxifying Molecules Are Differentially Expressed in LTT Lines

**Figure 2:**
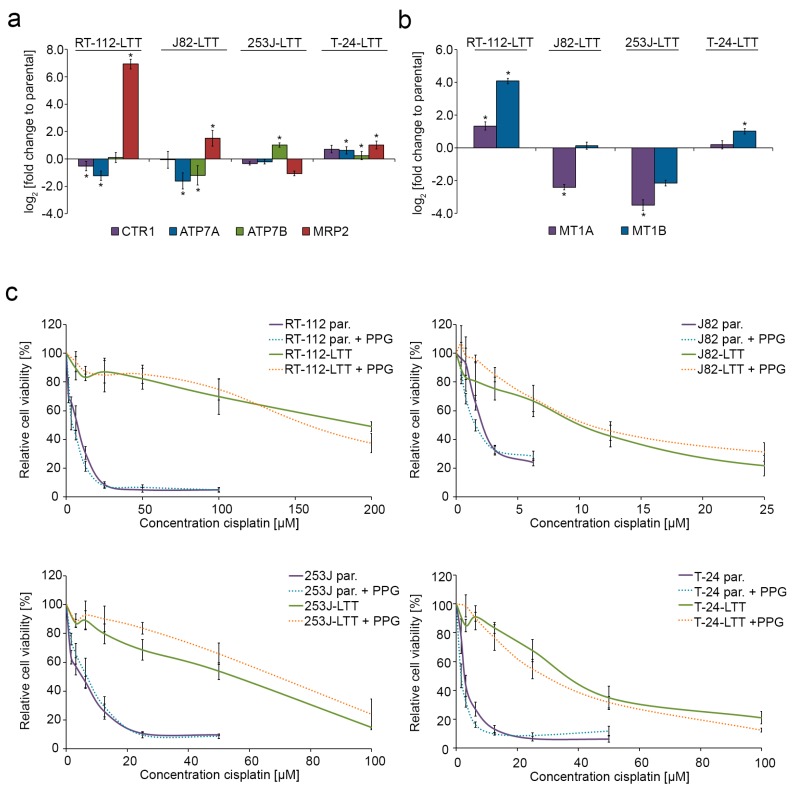
Cisplatin exporter and detoxifying molecules are differentially expressed in LTT lines. Relative fold change of (**a**) *CTR1*, *ATP7A*, *ATP7B*, *MRP2*, (**b**) *MT1A* and *MT1B* mRNA expression in RT-112-LTT, J82-LTT, 253J-LTT, T-24-LTT compared to their parental cell lines was measured by qRT-PCR. Expression levels in the untreated parental UCCs were set as 1. For endogenous expression data of parental UCCs see Figure S1a,b. *SDHA* was used as a reference gene and relative expression was calculated by the 2^−ΔΔ*C*t^ method. * *p* < 0.05. (**c**) After concomitant treatment with dl-propargylglycine (PPG) and cisplatin for 72 h, viability was measured by MTT assay in parental UCCs and LTTs. Untreated cells were set as 100. Values represent the mean ± SD of two independent experiments.

To analyse pre-target resistance as a potential mechanism in LTTs, we measured the mRNA expression of cisplatin transporters and detoxifying molecules. Cisplatin importer *CTR1* and the exporters *ATP7A* and *ATP7B* were mainly upregulated in T24-LTT compared to its parental cell line (Figure 2a, Figure S1a, Table S2). *ATP7B* was also significantly upregulated in 253J-LTT. Strikingly, mRNA expression of MRP2, which exports cisplatin glutathione conjugates, was strongly upregulated in RT-112-LTT, J82-LTT, and T24-LTT (Figure 2a, Table S2). Metallothionein mRNA expression was also significantly upregulated in two of four LTTs, but especially *MT1B* was downregulated in the two others (Figure 2b, Figure S1b, Table S2). Accordingly, some of the LTTs were co-resistant to CdCl_2_, ZnCl_2_, and to a lesser extent to H_2_O_2_ (Table S3). Thus, we investigated whether inhibition of metallothioneins by dl-propargylglycine (PPG, Table S4) sensitised LTTs to cisplatin. Concomitant treatment with IC_50_ values of PPG and cisplatin did however not significantly affect cisplatin sensitivity in either parental UCCs or LTT lines (Figure 2c).

Of note, we have previously reported that several other factors involved in cisplatin and glutathione metabolism, which are NRF2 targets, are also upregulated to different extents in the LTT lines, most prominently in RT-112-LTT and T24-LTT [16]. These data indicate that a number of different pre-target factors are implicated to various extents in cisplatin resistance in different sublines.

### 2.3. DNA-Cisplatin Adduct Formation and Extent of DNA Damage Is Reduced in LTTs

**Figure 3:**
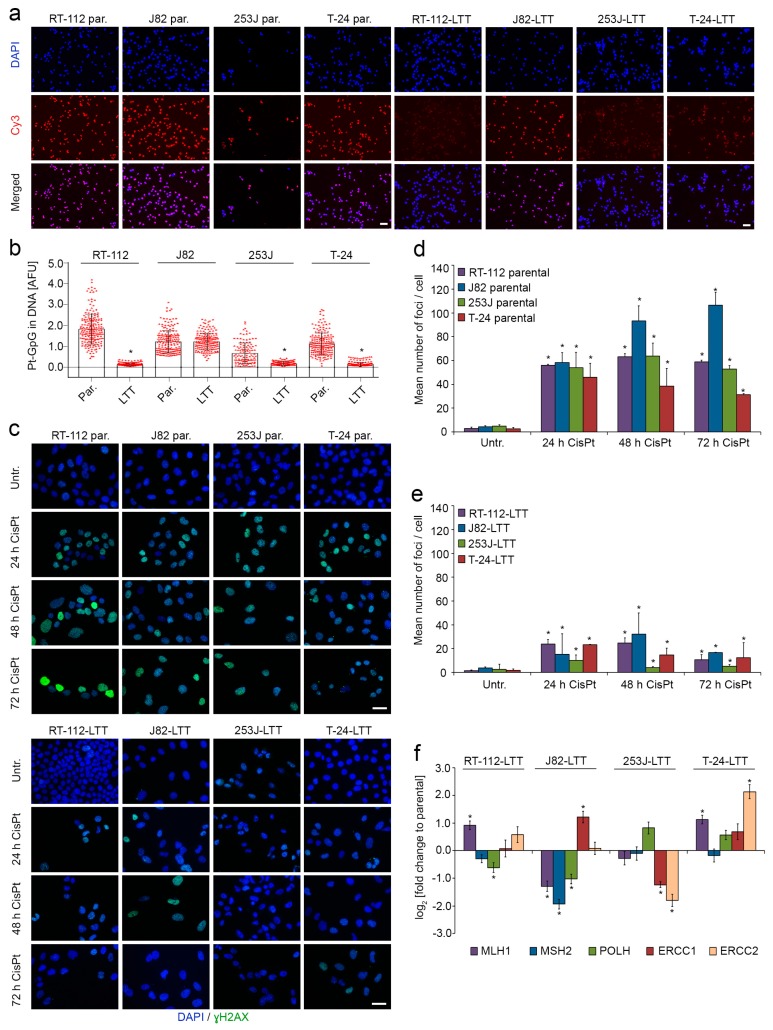
DNA-cisplatin adduct formation and extent of DNA damage are reduced in LTTs. (**a**) Representative immunofluorescence staining for Pt-adducts in parental UCCs and LTTs treated with 50 µM cisplatin for 4 h. Scale bar, 100 µm; (**b**) Quantification of Pt-adducts by immunofluorescent staining in parental UCCs and LTTs treated with 50 µM cisplatin for 4 h; (**c**) Representative immunofluorescence staining for pH2A.X (Ser139) foci in 24 h, 48 h, and 72 h IC_50_ or maintenance cisplatin-treated parental UCCs and LTTs, respectively, compared to their untreated controls. Scale bar, 100 µm; Quantification of pH2A.X (Ser139) foci by immunofluorescent staining in UCCs (**d**) and LTTs (**e**) treated with IC_50_ or maintenance cisplatin concentration, respectively, for 24, 48, and 72 h. Values represent the mean ± SD of biological duplicates; (**f**) Relative fold change of *MLH1*, *MSH2*, *POLH*, *ERCC1*, and *ERCC2* mRNA expression in LTTs was compared to their parental cell lines. Expression levels in the untreated parental cells were set as 1. For endogenous expression data of parental UCCs see Figure S1c. *SDHA* was used as a reference gene and relative expression was calculated by the 2^−ΔΔ*C*t^ method. Values represent the mean ± SD of three independent experiments. * *p* < 0.05, compared to untreated ctrl.

To investigate the role of on-target resistance mechanisms, parental UCCs and LTTs were treated with 50 µM cisplatin for 4 h and the amount of Pt-adducts was quantified (Figure 3a,b). Quantification revealed significantly fewer Pt-adducts in all LTTs except J82-LTT compared to their parental cell lines (Figure 3b). 

Concurringly, the number of γH2AX foci indicating DNA double-strand breaks originating from Pt-adducts was strongly increased after IC_50_ cisplatin treatment of parental UCCs (Figure 3c,d), with approximately 50 foci per cell in all cell lines after 24 h. Over time, the number of foci remained rather stable, with a slight but significant decrease in T-24 cells after 72 h and a significant increase in J82 cells. These differences in the time course may reflect differences in the DNA repair capacity of the cells. In contrast, in all four LTTs the increase in γH2AX-foci induced by maintenance concentration cisplatin treatment (at least 1.8-fold higher than parental IC_50_ doses) was much less pronounced (about three-fold less), 253J-LTT displaying the lowest level of DNA damage. Moreover, in all LTT lines except J82-LTT, the number of foci per cell was decreased after 72 h (Figure 3c–e). Therefore, cisplatin induces less DNA damage in LTTs, which may be more easily repaired.

Further, we measured mRNA expression of relevant components of the DNA repair machinery (*MLH1*, *MSH2*, *POLH*, *ERCC1*, and *ERCC2*; Figure 3f). In RT-112-LTT *MLH1* mRNA was significantly upregulated, while *POLH* was downregulated. Decreased mRNA expression of *MLH1*, *MSH2*, and *POLH* was detected in J82-LTT, while *ERCC1* expression was significantly increased. *ERCC1* and *ERCC2* mRNA expression were downregulated in 253J-LTT. In T-24-LTT *MSH2* was the only mRNA not differentially expressed, *MLH1* and *ERCC2* were strongly increased compared to the parental cell line (Figure 3f, Figure S1c, Table S2). Thus, several significant changes were detected in the expression of DNA damage repair factors, which were however not uniform between the individual LTT lines. 

### 2.4. LTTs Are More Resistant to Cisplatin-Induced Apoptosis

**Figure 4:**
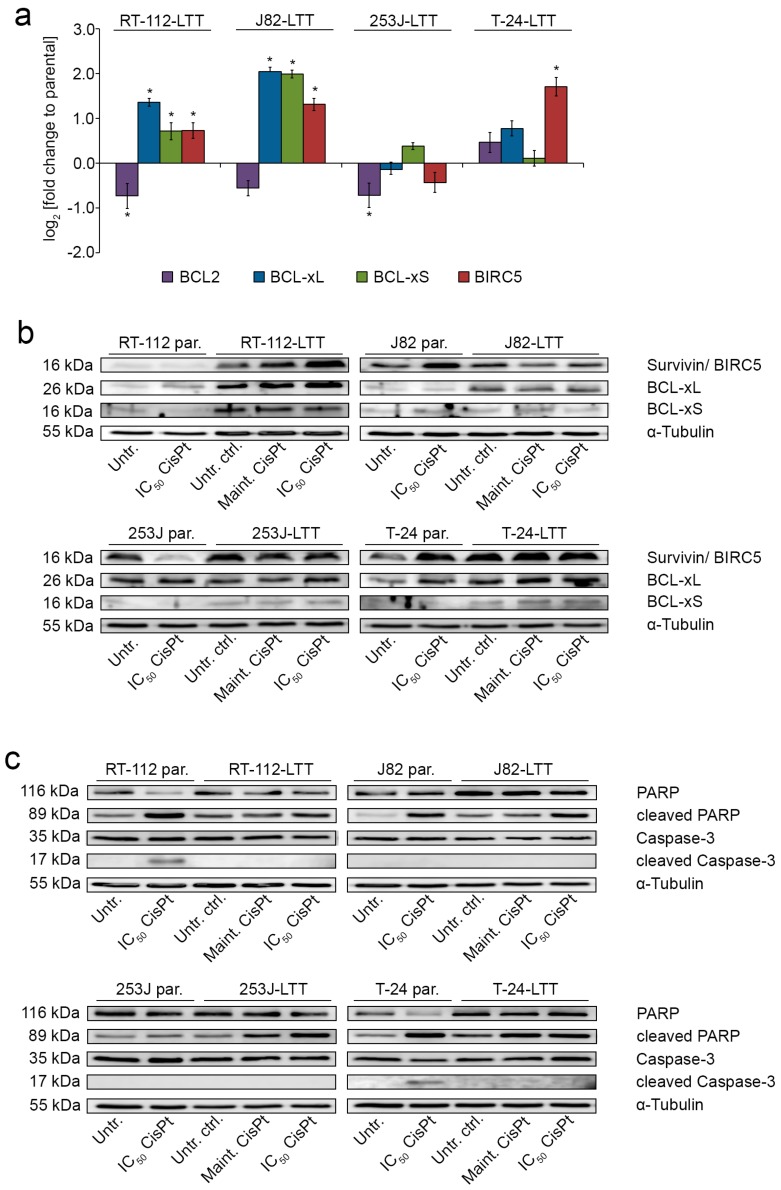

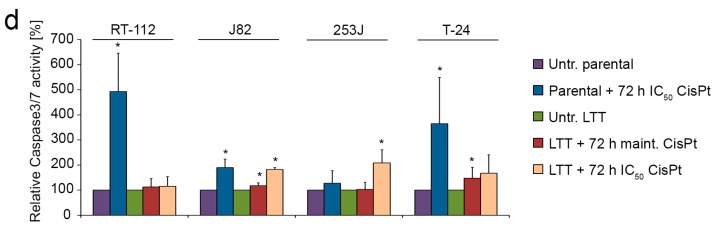
LTTs are more resistant to induction of cisplatin-induced apoptosis. (**a**) Relative fold change of *BCL2*, *BCL-xL*, *BCL-xS*, and *BIRC5*/Survivin mRNA expression in LTTs compared to their parental cell lines was measured by qRT-PCR. Expression levels in the untreated parental cells were set as 1. For endogenous expression data of parental UCCs see Figure S1d. *SDHA* was used as a reference gene and relative expression was calculated by the 2^−ΔΔ*C*t^ method. Values represent the mean ± SD of three independent experiments. BIRC5/Survivin, BCL-xL, BCL-xS (**b**), PARP, cleaved PARP, Caspase-3, and cleaved Caspase-3 (**c**) protein expression was detected in either untreated or IC_50_ doses treated parental UCCs and compared with LTTs under either untreated conditions or treated with maintenance or IC_50_ cisplatin concentration for 72 h. As a loading control, α-Tubulin was detected. One representative immunoblot is shown from two independent experiments. (**d**) Caspase 3/7 activity was measured in parental UCCs untreated or treated with IC_50_ cisplatin concentration and LTTs treated with maintenance or IC_50_ cisplatin concentration for 72 h. Values were adjusted to cell viability as measured by CellTiter Glo. Values represent the mean ± SD of two independent experiments. * *p* < 0.05.

Next, post-target resistance mechanisms preventing induction of apoptosis by cisplatin were studied in LTTs. The mRNA expression of the apoptotic regulators *BCL-xL*, *BCL-xS*, and *BIRC5*/Survivin were elevated in all LTTs except 253J-LTT. *BCL2* mRNA was slightly diminished in RT-112-LTT and 253J-LTT (Figure 4a, Figure S1d, Table S2). These results were verified on the protein level. In particular, short-term cisplatin treatment of parental cell lines increased Survivin protein in J82 and T-24, whereas a decrease was observed in 253J (Figure 4b, see Figure S2a for quantified expression). In all untreated LTT lines the endogenous protein expression of Survivin was increased compared to untreated parental cell lines (Figure S2). Following cisplatin treatment at maintenance or IC_50_ concentrations Survivin protein expression was further increased in RT-112-LTT (Figure 4b, Figure S2a). BCL-xL protein expression was slightly increased after short-term cisplatin treatment of some parental UCCs (Figure S2b, Figure 4b). Untreated LTTs, except 253J, displayed elevated levels of BCL-xL protein compared to parental UCCs, which were further increased upon cisplatin treatment in all LTTs except J82 (Figure 4b, Figure S2b).

Apoptotic responses of the cell lines to cisplatin-induced stress were followed on the molecular level via Poly (ADP-ribose) polymerase (PARP) and Caspase 3 cleavage (Figure 4c). PARP cleavage was induced in cisplatin treated RT-112, J82, and T-24 parental UCCs. A slight increase was also detected in all four LTTs, but only after treatment with their much higher respective IC_50_ cisplatin doses (Figure 4c). Caspase-3 cleavage was only detectable in IC_50_ cisplatin treated RT-112 and T-24 UCCs, but not in LTTs under any condition (Figure 4c). Accordingly, caspase-3/7 activity was strongly increased in parental RT-112, J82, and T-24 UCCs treated with IC_50_ cisplatin concentrations for 72 h, but much less in LTTs, even after treatment with their much higher IC_50_ dosages (Figure 4d).

**Figure 5:**
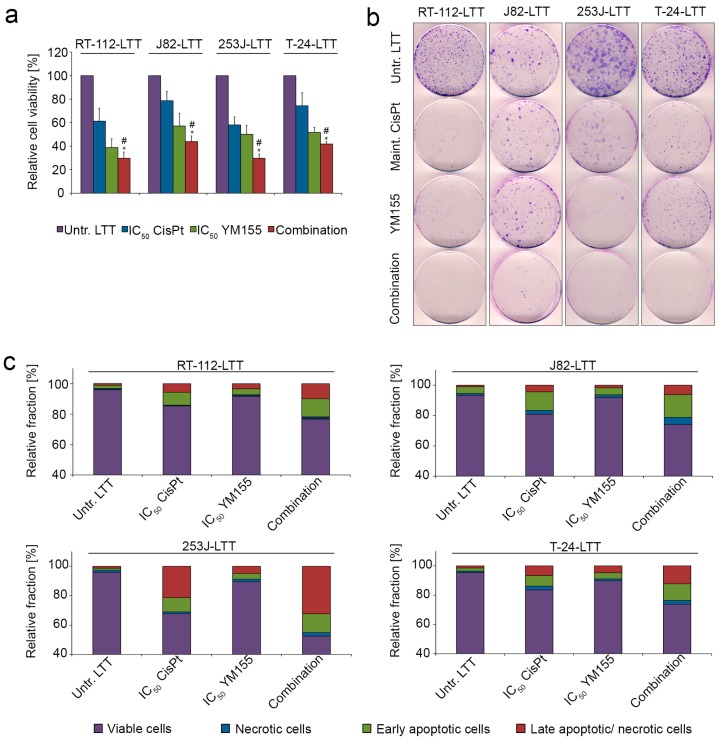

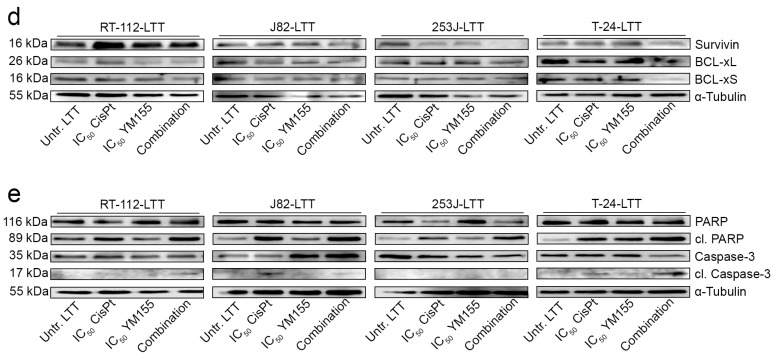
Pharmacological inhibition of Survivin by YM155 sensitised LTTs to cisplatin. (**a**) Cell viability was measured in untreated LTTs (set as 100) compared to LTTs treated with IC_50_ concentration of cisplatin, IC_50_ concentration of YM155, and the combination of both after 72 h by MTT assay. Values represent the mean ± SD of two independent experiments. * *p* < 0.05 CisPt vs. combination, # *p* < 0.05 YM155 vs. combination. Corresponding to combination indexes (CI) displayed in Table S5; (**b**) Colony formation assay of untreated LTTs compared to LTTs treated for 72 h with maintenance cisplatin concentration, IC_50_ concentration of YM155, or the combination. Cell clones were stained by Giemsa; (**c**) Induction of apoptosis and necrosis was analysed in untreated LTTs and those treated with IC_50_ concentration of cisplatin, IC_50_ concentration of YM155, or the combination of both after 72 h by combined Annexin V and PI staining with subsequent flow cytometry as shown in a bar graph; (**d**,**e**) Survivin, BCL-xL, BCL-xS (**d**), PARP, cleaved PARP, Caspase-3, and cleaved Caspase-3 (**e**) protein expression was detected in LTTs treated with IC_50_ cisplatin concentration, IC_50_ YM155 concentration, and the combination of both after 72 h compared to their untreated controls. As a loading control, α-Tubulin was detected. One representative immunoblot is shown from two independent experiments.

To directly investigate whether the increase in Survivin might provide a target for reversing resistance in the LTTs, we employed pharmacological inhibition of Survivin by YM155 (sepantronium bromide). Following the determination of its IC_50_ values in UCCs and LTTs (Table S4), we studied the effect of combined cisplatin and YM155 IC_50_ concentrations in LTTs. Interestingly, the combination further decreased cell viability, as measured by MTT assay, compared to the single treatments (Figure 5a). However, analysis by the Chou–Talalay method indicated at most additive, but not synergistic actions of cisplatin and YM155 (Table S5). Analogous results were obtained for their clonogenic potential (Figure 5b) as well as for the induction of apoptosis, as determined by the percentage of apoptotic cells (Figure 5c, Figure S3a). Concomitantly, Survivin protein expression was decreased after combined treatment with cisplatin and YM155 in RT-112-LTT, 253J-LTT, and T-24-LTT compared to treatment with cisplatin only. BCL-xL and BCL-xS protein expression was mostly unaffected by YM155, except in T-24-LTT cells (Figure 5d). PARP cleavage was observed after cisplatin single treatment, but more prominently in the combination with YM155 (Figure 5e, Figure S3b). Cleaved Caspase-3 was detected in RT-112-LTT and T-24-LTT concomitantly treated with cisplatin and YM155 (Figure 5e).

## 3. Discussion

Resistance to platinum-based treatment is a major limitation of chemotherapy. To elucidate mechanisms underlying cisplatin resistance in UC and to identify new molecular targets for combined therapeutic approaches, we comprehensively characterised long-term cisplatin treated urothelial carcinoma cell lines for their proliferation capacity, alterations in cisplatin uptake, efflux, and detoxification as well as for changes in DNA repair and regulation of apoptosis, in accord with the proposed pre-, on-, post-, and off-target resistance mechanisms [8].

At cisplatin doses two- to eight-fold higher than the IC_50_ doses of treatment naïve parental cells, LTTs managed to evade apoptosis and recovered over time to re-enter the cell cycle. Several findings of the present and former studies indicate a contribution of pre-target mechanisms to cisplatin resistance in LTTs. Notably, the *MRP2* efflux transporter mediating cisplatin extrusion after its conjugation to GSH was strongly overexpressed in three of four LTTs. Previously, we measured increased GSH levels in most LTTs in our previous study [16], suggesting that a predominant mechanism of cisplatin resistance in UC is based on conjugation of the drug with GSH allowing increased efflux via MRP2.

Apart from GSH-conjugates, cisplatin can also be detoxified by binding to metallothioneins. Nevertheless, J82 did not show any difference in the amount of Pt-adducts between parental and LTT cells despite increased *MT1B* levels. However, we found metallothioneins mainly overexpressed in RT-112-LTT, suggesting that these cells evade cisplatin-induced stress by conjugation of the drug to metallothioneins.

Upregulation of *ATP7* transmembrane carriers exporting unconjugated cisplatin was only observed in individual LTTs, suggesting a biologically irrelevant and minor role in cisplatin resistance. Likewise, we observed in one cell line only a decreased expression of the *CTR1* importer, although a positive correlation had been found between CTR1 expression and pathological outcome in Pt-treated muscle-invasive bladder cancer [34].

As expected from the activation of pre-target mechanisms like increased detoxification and efflux, we observed a lower platinum adduct burden and less DNA damage in LTTs than in their parental cell lines. Conceivably, LTTs may sustain a more limited degree of DNA damage which they can manage to repair over time. In addition, we found evidence for changes in expression of critical DNA repair factors that may help escaping from the selective pressure of cisplatin. Across the LTT lines various DNA repair factors were upregulated. T-24-LTT displayed upregulation of several factors with strong overexpression of *ERCC2*, whereas the MMR genes were significantly downregulated in J82-LTT, potentially diminishing futile repair cycles and the triggering of apoptosis [35]. In summary, while a complete characterisation of DNA repair in the LTTs has not yet been performed, our data suggest that on-target mechanisms resulting from increased activity of the DNA repair machinery could make an additional contribution to the major effects of pre-target resistance mechanisms in LTTs.

As evasion of apoptosis is a main mechanism of post-target resistance, we determined the expression profile of several key apoptosis-regulating factors in LTTs. Our investigation revealed increased mRNA and protein expression of anti-apoptotic BCL-xL and *BIRC5*/Survivin in most LTTs. Specifically in RT-112-LTT, elevated Survivin protein expression might significantly contribute to cisplatin resistance. Concurringly, PARP and Caspase-3 cleavage as markers of apoptosis were detected in short-term cisplatin treated parental UCCs, but not in LTTs at maintenance cisplatin concentrations, and even only to a limited degree following treatment with IC_50_ cisplatin doses. These observations indicate a substantial contribution of suppression of apoptosis by upregulation of anti-apoptotic factors to cisplatin resistance in LTTs. In particular, our findings are in accord with a number of publications identifying Survivin as a crucial anti-apoptotic factor in bladder cancer [31,32]. Interestingly, simultaneous siRNA-mediated knockdown of BCL-xL and Survivin sensitised the bladder cancer cell lines EJ28 and J82 to cisplatin [36]. We pursued pharmacologic inhibition of Survivin for a combination treatment approach. YM155 inhibits transcription of Survivin by binding to the transcription factor ILF3/NF110 or disrupting the ILF3/p54^nrb^ complex [37] and has been demonstrated to significantly diminish *BIRC5*/Survivin mRNA and protein expression without affecting other inhibitors of apoptosis (IAPs) or the BCL2 family member MCL-1 [38]. In this fashion, YM155 reverted resistance in cells from different cancer types [38,39,40]. In the UC LTTs, combined treatment with YM155 and cisplatin decreased clonogenic potential and further increased apoptosis, albeit not in a synergistic manner. Since RT-112-LTT displayed the most significant induction of Survivin expression upon cisplatin treatment, upregulation of anti-apoptotic factors like Survivin may be one major resistance mechanism of this subline. Concurringly, these cells were particularly sensitised to cisplatin by YM155. Decreased cell viability and colony numbers have also been reported in 5637 and T-24 UCC cells treated with YM155. In that study, increased Survivin expression was ascribed to NF-κB activation [41]. However, in our previous study on LTTs, we did not observe changes in *RELA*/NF-κB p65 mRNA and protein expression [16] and we have not observed induction of Survivin by NF-κB activation in several UCCs (I. Müller, W. A. Schulz, unpublished observations). Therefore, other factors may account for the increased protein expression of Survivin and BCL-xL in LTTs. However, results of our study highlight anti-apoptotic factors like Survivin as promising targets for combination therapy approaches to overcome evasion of apoptosis and finally chemoresistance.

As an example for off-target mechanisms, we demonstrated in a previous study that epithelial–mesenchymal transition evidently contributes to cisplatin resistance in the UC LTT lines [33]. However, cisplatin resistance did not originate from selection for intrinsically resistant cells with stem cell-like properties marked by CD90/CK14 [33].

Finally, the data from our comprehensive study across four pairs of UCCs sensitive or resistant to cisplatin provides evidence for the multifaceted character of cisplatin resistance and the diversity of mechanisms that might also apply to UC patients. Obviously, cellular stress induced by cisplatin treatment in cancer cells selects for more than one mechanism of drug resistance. Recently, we also observed increased activation of autophagy in LTTs, which has an important function in regulation of metabolic substrates like e.g., GSH and intracellular reactive oxygen species (ROS) removal. Thus, activation of autophagy and oxidative stress resistance mediated by glucose metabolism [16,42] appear to be further mechanisms contributing to cisplatin resistance. Inhibition of autophagy, which may interfere with glucose metabolism, therefore, reduces the efficiency of intracellular ROS removal [16,43]. Accordingly, LTTs could be sensitised to cisplatin by autophagic inhibitors, such as chloroquine, 3-methyladenine, and SAR405 [44].

To decrease the reaction with diverse metabolic biomolecules, such as the thiol-containing metallothionein proteins and GSH, and facilitate DNA binding, new designs for covalently binding platinum drugs are desirable. Therefore, 9-aminoacridine Pt-complexes [45], hybrid compounds combining biologically active nitroxyl radicals and platinum pharmacophores [46], or other platinum intercalators with high anti-cancer activity, such as multinuclear complexes with active ligands were developed to increase cellular cisplatin uptake, lower the reactivity with the aforementioned metabolic biomolecules, and elevate interstrand crosslinking [47,48]. Further in vitro and in vivo studies are necessary to validate their efficiency in the future.

In conclusion, each of the UC cell lines presented an individual assortment of mechanisms acting at the pre-, on- and post-target level. Predominant mechanisms appear to be detoxification by conjugation and efflux together with enhanced evasion of apoptosis. The results obtained with inhibitors of Survivin and metallothioneins moreover suggest that pharmacological inhibition strategies targeting only one mechanism at a time may enhance sensitivity towards cisplatin treatment only to a limited extent. Thus, future studies should also explore whether combination therapies concomitantly targeting multiple mechanisms underlying cisplatin resistance are a realistic option.

## 4. Materials and Methods

### 4.1. Cell Culture and Treatment

The human UCCs RT-112, T-24, 253J, and J82 were grown in DMEM GlutaMAX-I (Gibco, Darmstadt, Germany) containing 10% FCS. LTTs were generated by adding *cis*-diamminedichloroplatinum-II (Cisplatin; Accord Healthcare, London, UK) after every passage at escalating doses over months [33]. Generated LTTs were cultivated under maintenance cisplatin doses of 50, 23, 6.6, or 3.3 µM cisplatin, respectively (see Figure 1). Respective IC_50_ doses of the LTTs were 210, 30, 50, or 10 µM. For short-term treatment (STT) of treatment naïve UCCs, cisplatin was applied for 72 h at IC_50_ doses of 10, 3, 3.5, or 1.8 µM, respectively. Cadmium chloride (#655198), zinc chloride (#Z0152), hydrogen peroxide solution (#216763-M), and dl-propargylglycine (#P7888) were purchased from Sigma-Aldrich (St. Louis, MO, USA). Doxorubicin (#324380), gemcitabine (#S1149), and YM155 (#S1130) were purchased from Calbiochem (Merck, Darmstadt, Germany) and Selleck Chemicals (Munich, Germany), respectively. Combination indexes (CI) were calculated by the Chou–Talalay method using CompuSyn software, version 1.0 [49].

### 4.2. Measurements of Cell Viability, Clonogenicity, and Proliferation

Cell viability was measured by MTT assay (Sigma-Aldrich, St. Louis, MO, USA). For colony formation assay cells were seeded at low density, maintained for 2 weeks and stained with Giemsa (Merck, Darmstadt, Germany). Cell proliferation was measured by EdU incorporation assay (baseclick GmbH, Neuried, Germany).

### 4.3. Molecular Analyses

RNA isolation, cDNA synthesis, and quantitative real-time PCR were performed as previously described [33]. Gene expression was determined using self-designed primers on the Lightcycler 96 system (Roche, Basel, Switzerland) (Table S6). The housekeeping gene *SDHA* was used for normalization.

### 4.4. Measurement of Caspase 3/7 Activity

Caspase-Glo 3/7 Assay and CellTiter-Glo Luminescent Cell Viability Assay (Promega, Fitchburg, WI, USA) were performed according to the manufacturer’s protocols. Relative Caspase 3/7 activity was normalised against cell viability.

### 4.5. Immunofluorescence

Immunofluorescence staining was performed as previously described [16]. Quantification of pH2A.X Ser139-Foci was performed using the *Focinator* software tool [50].

### 4.6. Quantification of Pt-(GpG) Adducts

The levels of Pt-adduct formation in the nuclear DNA of individual cells were determined by an immuno-cytological assay as previously described [51]. In brief, exponentially growing cells were exposed to cisplatin (Accord Healthcare, London, UK; 50 µM in culture medium) for 4 h at 37 °C. Cells were detached and spotted onto microscopic adhesion slides (Superfrost Plus Gold, ThemoScientific), air dried, fixed in methanol (−20 °C, 30 min), and denatured by alkaline treatment (60% 70 mM NaOH/140 mM NaCl and 40% methanol) for 5 min at 0 °C. Slides were further digested successively with pepsin and proteinase K (400 µg/mL, each for 10 min at 37 °C). Pt-(GpG) adducts in DNA were immuno-stained with Mab R-C18 (stock: Thomale lab, Institute for Cell Biology, Essen; 0.01 µg/mL; 4 °C, overnight) and visualised with Cy3-labelled rabbit anti-(rat Ig) antibody (#312-165-003, Dianova, Hamburg, Germany). Nuclear DNA was counterstained with DAPI (1 µg/mL in PBS). DAPI- and Cy3-derived signals from individual cell nuclei were integrated and measured separately using a microscope-coupled digital image analysis system (Zeiss Axioplan, Göttingen, Germany; ACAS 6.0 Image Analysis System, Ahrens Electronics, Bargteheide, Germany). Relative adduct concentrations per cell were calculated by dividing antibody-derived values by DAPI values of each nucleus and are expressed as Arbitrary Fluorescence Units (AFU). Values were calculated as means of at least 100 measured cells per sample, error bars represent 95% confidence intervals.

### 4.7. Western Blot Analysis

Determination of protein concentration and immunodetection of proteins was performed as described [16] using antibodies listed in Table S7.

### 4.8. Flow Cytometry

Cell-cycle analyses of cisplatin treated parental UCCs and LTTs were performed after 72, 168, and 240 h by staining attached and supernatant cells with PI buffer containing 50 mg/mL propidium iodide (PI), 0.1% sodium citrate, and 0.1% Triton X-100 [52]. Assessment of apoptotic cell death was additionally determined by Annexin V/PI staining as described previously [16]. All analyses were determined using the MACSQuant flow cytometer with the MACSQuant Analyzer 10 software (Miltenyi Biotec GmbH, Bergisch Gladbach, Germany).

## Supplementary Materials

Supplementary materials can be found at http://www.mdpi.com/1422-0067/19/2/590/s1.

## Abbreviations

UCC	Urothelial carcinoma cell line
SDHA	Succinate dehydrogenase complex
CTR1	Solute carrier family 31 member 1
ATP7A	ATPase copper transporting alpha
ATP7B	ATPase copper transporting beta
MT	Metallothionein
MRP2	Multi-drug resistance protein 2
ERCC1	ERCC excision repair 1, Endonuclease non-catalytic subunit
ERCC2	ERCC excision repair 2, TFIIH core complex helicase subunit
MLH1	MutL homolog 1
MSH2	MutS homolog 2
POLH	DNA polymerase eta
BCL-2	B-Cell CLL/Lymphoma 2
BCL-XL	BCL2 like 1
BIRC5	Baculoviral IAP repeat containing 5
γH2AX	Phosphorylated H2A histone family member X
Pt-adduct	Platinum-adduct
