# Supplementary material for: Multifaceted Mechanisms of Cisplatin Resistance in Long-Term Treated Urothelial Carcinoma Cell Lines
Source: Int J Mol Sci. 2018 Feb 16;19(2):590. doi: 10.3390/ijms19020590 (PMC5855812; doi:10.3390/ijms19020590)
Supplement: Supplementary file 1 [file ijms-19-00590-s001.pdf]

Supplementary Material: Multifaceted Mechanisms of Cisplatin Resistance in Long-Term Treated Urothelial Carcinoma Cell Lines

Margaretha A. Skowron, Margarita Melnikova, Joep G. H. van Roermund, Andrea Romano, Peter Albers, Jürgen Thomale, Wolfgang A. Schulz, Günter Niegisch and Michèle J. Hoffmann

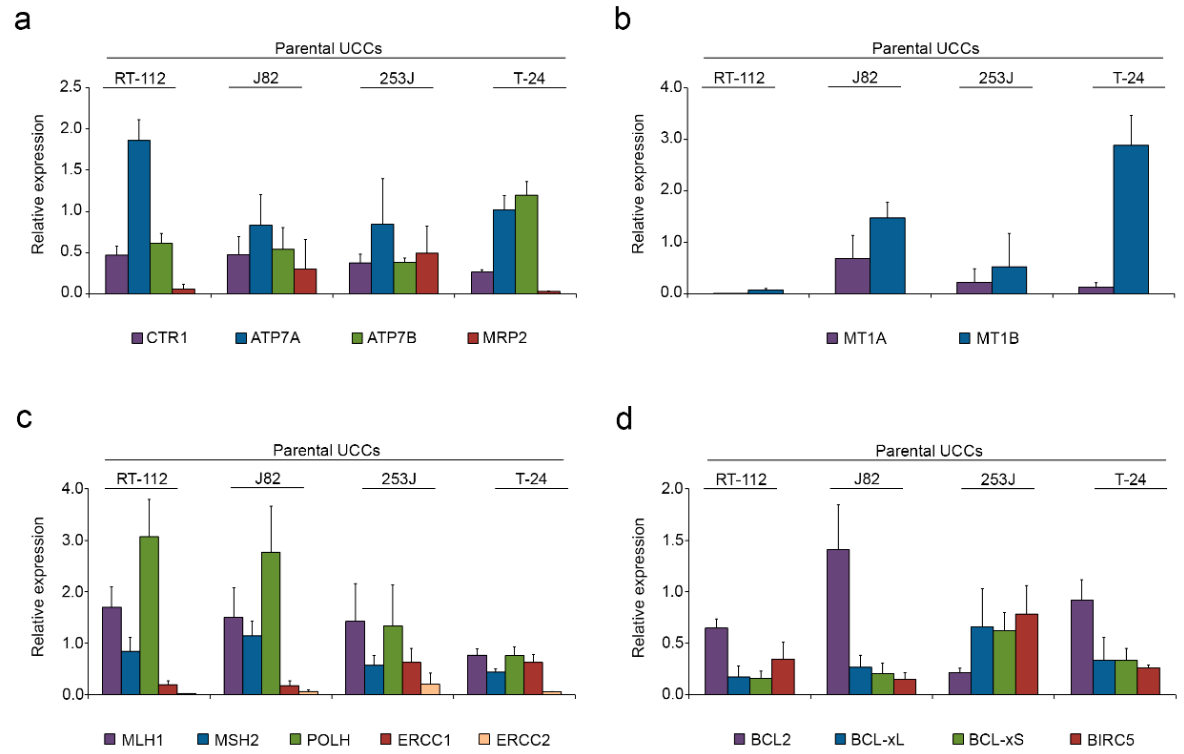

Figure S1. Potential factors involved in cisplatin resistance. Relative mRNA concentration of (**a**) *CTR1*, *ATP7A*, *ATP7B*, *MRP2*, (**b**) *MT1A*, *MT1B*, (**c**) *MLH1*, *MSH2*, *POLH*, *ERCC1*, *ERCC2*, d) *BCL2*, *BCL-xL*, *BCL-xS*, and *BIRC5* in untreated parental UCCs RT-112, J82, 253J, and T-24 was measured by qRT-PCR. *SDHA* was used as a reference gene. Values represent the mean ± SD of biological triplicates.

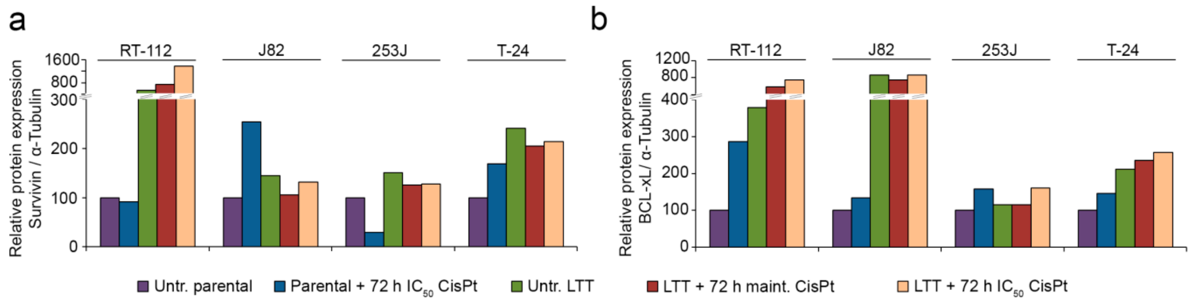

Figure S2. Increased Survivin protein expression and influence of YM155 on cleaved PARP protein expression in LTTs. Normalised quantification of (**a**) Survivin and (**b**) BCL-xL protein expression detected in parental UCCs treated with IC_{50} cisplatin concentration and LTTs treated with maintenance or IC_{50} cisplatin concentration for 72 h compared to their untreated controls corresponding to the data in Figure 4b. Untreated parental UCCs were set as 100. As a loading control, α-Tubulin was detected.

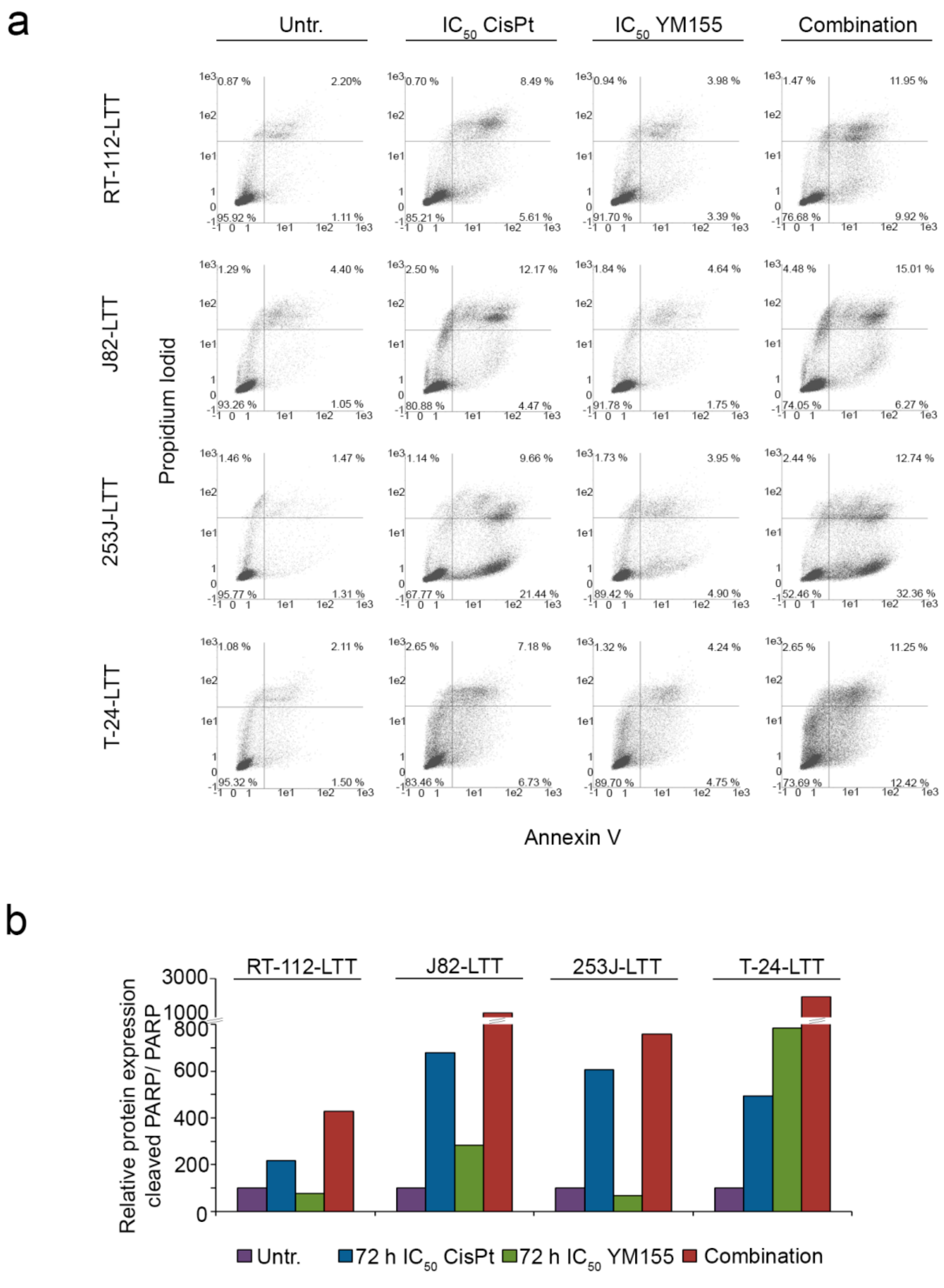

Figure S3. Pharmacological inhibition of Survivin by YM155 sensitised LTTs to cisplatin. Induction of necrosis and apoptosis was analysed in LTTs treated with IC_{50} concentration cisplatin, IC_{50} concentration YM155, and in the concomitant combination of both after 72 h by (**a**) combined Annexin V and PI staining with subsequent flow cytometry. The percentages in the figure indicate viable cells (bottom left), necrotic cells (top left), early apoptotic cells (bottom right), and late apoptotic/ necrotic cells (top right). Values represent the mean of biological duplicates. (**b**) Normalised quantification of cleaved PARP protein expression detected in LTTs treated with IC_{50} cisplatin, IC_{50} YM155 concentration and in combination of both for 72 h compared to their untreated LTTs corresponding to the data in Figure 5e. Untreated LTTs were set as 100. As a loading control, α-Tubulin was detected.

Table S1. Cross-resistance of LTTs to other chemotherapy compounds. IC_{50} values of gemcitabine and doxorubicin treated LTTs and their parental cell lines after 72 h treatment. Cross-resistance was defined as IC_{50} LTT subline / IC_{50} respective parental cell line > 2. * $p < 0.05$

	**Gemcitabine [µM]**	**Doxorubicin [nM]**
RT-112	6.3 ± 1.5	81.7 ± 10.4
RT-112-LTT	106.6 ± 11.5	115.0 ± 13.2
Resistance factor	**(16.92)** *	**(1.40)** *
J82	22.5 ± 3.5	131.7 ± 16.1
J82-LTT	31.5 ± 4.9	75.5 ± 5.1
Resistance factor	(1.40)	**(0.57)** *
253J	7.1 ± 2.6	115.0 ± 7.1
253J-LTT	5.3 ± 1.5	137.5 ± 10.6
Resistance factor	(0.75)	(1.19)
T-24	6.7 ± 1.5	52.8 ± 3.9
T-24-LTT	11.3 ± 1.2	112.5 ± 17.7
Resistance factor	**(1.70)** *	**(2.13)** *

Table S2. Summary of qRT-PCR data. Up- (arrows upwards), downregulated (arrows downwards), and unchanged (−) mRNA levels of several genes involved in pre-, on-, and post-target cisplatin mechanisms. * $p < 0.05$.

Pre-target					
**Factor**	**Function**	**RT-112/RT-112-LTT**	**J82/J82-LTT**	**253J/253J-LTT**	**T-24/T-24-LTT**
CTR1	Influx	↓ *	−	−	↑ *
ATP7A	Efflux	↓ *	↓ *	−	↑ *
ATP7B	Efflux	−	↓ *	↑ *	−
MRP2	Inactivation	↑ *	↑ *	−	↑ *
MT1A	Inactivation	↑ *	↓ *	↓ *	−
MT1B	Inactivation	↑ *	−	−	↑ *
On-target					
**Factor**	**Function**	**RT-112/RT-112-LTT**	**J82/J82-LTT**	**253J/253J-LTT**	**T-24/T-24-LTT**
MLH1	MMR	↑ *	↓ *	−	↑ *
MSH2	MMR	−	↓ *	−	−
POLH	TLS	↓ *	↓ *	−	−
ERCC1	NER	−	↑ *	↓ *	−
ERCC2	NER	−	−	↓ *	↑ *

Post-target					
**Factor**	**Function**	**RT-112/RT-112-LTT**	**J82/J82-LTT**	**253J/253J-LTT**	**T-24/T-24-LTT**
BCL2	Anti-apoptotic	↓ *	−	−	−
BCL-xL	Anti-apoptotic	↑ *	↑ *	−	−
BCL-xS	Anti-apoptotic	↑ *	↑ *	−	−
BIRC5	Anti-apoptotic	↑ *	↑ *	−	↑ *

Table S3. LTTs are co-resistant to various cytotoxic substances. IC_{50} values of cisplatin, cadmium chloride ($CdCl_2$), zinc chloride ($ZnCl_2$), or hydrogen peroxide (H_2O_2) treated LTTs and their parental cell lines after 72 h treatment. Cross-resistance was defined as IC_{50} LTT subline/IC_{50} respective parental cell line >2. * $p < 0.05$

	**Cisplatin [µM]**	**$CdCl_2$ [µM]**	**$ZnCl_2$ [µM]**	**H_2O_2 [µM]**
RT-112	12.1 ± 1.01	8.0 ± 1.3	240 ± 28.3	35 ± 14.1
RT-112-LTT	215 ± 21.2	17.8 ± 2.8	397.5 ± 31.8	61 ± 12.7
Resistance factor	**(17.77)** *	**(2.23)** *	**(1.65)** *	(1.74)
J82	1.9 ± 1.4	1.1 ± 0.2	185 ± 35.4	32.5 ± 3.5
J82-LTT	8.9 ± 0.1	3.0 ± 0.1	365 ± 21.2	23.5 ± 4.9
Resistance factor	**(4.69)** *	**(2.72)** *	**(1.97)** *	(0.72)
253J	3.7 ± 0.8	5.0 ± 0.5	250 ± 42.4	25 ± 7.1
253J-LTT	50.3 ± 6.7	18.0 ± 4.3	410 ± 14.1	40 ± 28.3
Resistance factor	**(13.59)** *	**(3.60)** *	**(1.64)***	(1.60)
T-24	2.8 ± 0.7	65 ± 5.0	190 ± 14.1	47.5 ± 10.6
T-24-LTT	28.8 ± 1.8	83.5 ± 7.4	430 ± 28.3	90 ± 28.3
Resistance factor	**(10.29)** *	**(1.28)** *	**(2.26)** *	(1.89)

Table S4. Determination of IC_{50} values to PPG and YM155. IC_{50} values of propargylglycine (PPG) or YM155 treated LTTs and their parental cell lines after 72 h.

	**PPG [mM]**	**YM155 [nM]**
RT-112	17.3 ± 6.7	8.3 ± 1.2
RT-112-LTT	13.8 ± 1.7	1.7 ± 0.5
J82	4.6 ± 1.2	10.1 ± 16.2
J82-LTT	4.7 ± 0.7	7.6 ± 1.1
253J	18.5 ± 4.9	31 ± 3.6
253J-LTT	5.3 ± 0.1	47.3 ± 2.5
T-24	7.9 ± 0.9	1.5 ± 0.5
T-24-LTT	10.5 ± 2.9	4.3 ± 0.5

Table S5. Combination indexes (CI) calculated for the combination of cisplatin (maintenance and IC_{50} concentration) and YM155 (IC_{50} concentration) in LTTs (CI < 1 indicates synergystic, = 1 indicates additive, and > 1 indicates antagonistic effect).

	**Maint. CisPt + YM155**	**IC_{50} CisPt + YM155**
RT-112-LTT	1.32	1.58
J82-LTT	1.59	1.47
253J-LTT	1.14	1.06
T-24-LTT	1.55	1.61

Table S6. Primer sequences for quantitative real-time-PCR. Sequences of primers (5′-3′) used for quantitative real-time-PCR including length of PCR product and annealing temperature. bp.: base pair; Fwd.: Forward; Rev.: Reverse.

Gene Name	Accession Number	Size [bp]		Sequence 5′-3′	$T_{Annealing}$ [°C]
SDHA	NM_004168	140	Fwd.	GCCAGGACCTAGAGTTTGTTCA	55
			Rev.	CTTTCGCCTTGACTGTTAATGA	
ATP7A	NM_000052	116	Fwd.	GCTACCTTGTCAGACACGAATGAG	57
			Rev.	TCTTGAACTGGTGTCATCCCTTT	
ATP7B	NM_001243182	117	Fwd.	TACCCATTGCAGCAGGTGTC	56
			Rev.	ACTTGAGCTGCAGGGATGAG	
BCL2	NM_000633	106	Fwd.	CCTGTGGATGACTGAGTACCTG	61
			Rev.	CAGAGGCCGCATGCTGGG	
BCL-xL	NM_138578	118	Fwd.	TAAACTGGGGTCGCATTGTG	58
			Rev.	AGGTAAGTGGCCATCCAAGC	
BCL-xS	NM_138578	138	Fwd.	GCAGTAAAGCAAGCGCTGAG	58
			Rev.	GTTCCACAAAAGTATCCTGTTCAAAG	
BIRC5	NM_001168	113	Fwd.	CTCAAGGACCACCGCATCT	55
			Rev.	TCGTTCTCAGTGGGGCAGT	
CTR1	NM_001859	167	Fwd.	GGGGATGAGCTATATGGACTCC	57
			Rev.	TCACCAAACCGGAAAACAGTAG	
ERCC1	NM_202001	77	Fwd.	AGGAAGAAATTTGTGATAC	50
			Rev.	TGTGTAGATCGGAATAAG	
ERCC2	NM_000400	100	Fwd.	CTGGAGGTGACCAAACTCATCTA	55
			Rev.	CCTGCTTCTCATAGAAGTTGAGC	
LGR5	NM_003667	101	Fwd.	ACAGGAAATCATGCCTTACAGAGCTT	58
			Rev.	ACTCCAAATGCACAGCACTGGT	
MLH1	NM_001167617	91	Fwd.	AAGTTGTTGGCAGGTATT	50
			Rev.	GGTAGTGTCCTAACATCAG	
MSH2	NM_000251	75	Fwd.	CTTCTTCTGGTTCGTCAGTATAGA	55
			Rev.	ATCATTCTCCTTGGATGCCTTAT	
MRP2	NM_000392	150	Fwd.	GCACCGACTATCCAGCATCTC	57
			Rev.	TCAGCAGTTGCTTGTGCAAGA	
MT1A	NM_005946	156	Fwd.	ACTGGTGGCTCCTGCACCTGCACT	61
			Rev.	ACAGCAGCTGCACTTCTCTGAT	
MT1B	NM_005947	105	Fwd.	CAAATGGATCCCAACTGCTC	55
			Rev.	GCAGCAGCACTTCTTGCAG	
POLH	NM_006502	170	Fwd.	GGATAGAATACATGGGTGAAC	52
			Rev.	TCCTGGGAAGTTCTTACTAC	
XAF1	NM_017523	133	Fwd.	GATCCACTTTTGATGTCAGAG	52
			Rev.	CCTGATGTTGATTTAGGATCG	

Table S7. Antibodies used for western blot analyses. Antibodies for western blotting of whole cell lysates, including specifications and dilutions.

Target	Size [kDa]	Dilution	Cat. Number	Company
Survivin	16	1:1000	2808	Cell Signaling, Cambridge, UK
PARP	116	1:1000	9532	Cell Signaling, Cambridge, UK
cleaved PARP	89	1:1000	9541	Cell Signaling, Cambridge, UK
Caspase-3	35	1:1000	9665	Cell Signaling, Cambridge, UK
cleaved Caspase-3	17	1:1000	9664	Cell Signaling, Cambridge, UK
Bcl-x	16, 26	1:2000	556361	BD Bioscience, San Jose, CA, USA
α-Tubulin	55	1:20000	ab4074	Abcam, Cambridge, UK
HRP-conjugated goat-anti-rabbit antibody	-	1:5000	sc-2004	Santa Cruz Biotechnology, Heidelberg, Germany
HRP-conjugated goat-anti-mouse antibody	-	1:5000	sc-2005	Santa Cruz Biotechnology, Heidelberg, Germany
